# Evaluation of the MVCT-based radiomic features as prognostic factor in patients with head and neck squamous cell carcinoma

**DOI:** 10.1186/s12880-023-01055-w

**Published:** 2023-08-01

**Authors:** Kota Abe, Noriyuki Kadoya, Kei Ito, Shohei Tanaka, Yujiro Nakajima, Shimpei Hashimoto, Yuhi Suda, Takashi Uno, Keiichi Jingu

**Affiliations:** 1 Department of Radiation Oncology, MR Linac ART Division, Graduate School of Medicine, Chiba University, 1-8-1 Inohana, Chuo-ku, Chiba, 260-8670 Japan; 2 Department of Radiation Oncology, Tokyo Metropolitan Cancer and Infectious Diseases Center Komagome Hospital, 3-18-22 Honkomagome, Bunkyo-ku, Tokyo, 113-8677 Japan; 3 Department of Radiation Oncology, Tohoku University Graduate School of Medicine, 1-1 Seiryo- machi, Aoba-ku, Sendai, 980-8574 Japan; 4 Department of Radiological Sciences, Komazawa University, 1-23-1 komazawa, Setagaya-ku, Tokyo, 154-8525 Japan; 5 Saitama Prefectural Cancer Center, 780 large section of a town Omuro, Ina-machi, Kitaadachi- gun, Saitama, 362-0806 Japan; 6 Diagnostic Radiology and Radiation Oncology, Graduate School of Medicine, Chiba University, 1-8-1 Inohana, Chuo-ku, Chiba, 260-8670 Japan

**Keywords:** Radiomics, Head and neck cancer, Megavoltage computed tomography (MVCT), Radiotherapy, Machine learning

## Abstract

**Background:**

Megavoltage computed tomography (MVCT) images acquired during each radiotherapy session may be useful for delta radiomics. However, no studies have examined whether the MVCT-based radiomics has prognostic power. Therefore, the purpose of this study was to examine the prognostic power of the MVCT-based radiomics for head and neck squamous cell carcinoma (HNSCC) patients.

**Methods:**

100 HNSCC patients who received definitive radiotherapy were analyzed and divided into two groups: training (n = 70) and test (n = 30) sets. MVCT images obtained using TomoTherapy for the first fraction of radiotherapy and planning kilovoltage CT (kVCT) images obtained using Aquilion LB CT scanner were analyzed. Primary gross tumor volume (GTV) was propagated from kVCT to MVCT images using rigid registration, and 107 radiomic features were extracted from the GTV in MVCT and kVCT images. Least absolute shrinkage and selection operator (LASSO) Cox regression model was used to examine the association between overall survival (OS) and rad score calculated for each patient by weighting the feature value through the coefficient when features were selected. Then, the predictive values of MVCT-based and kVCT-based rad score and patient-, treatment-, and tumor-specific factors were evaluated.

**Results:**

C-indices of the rad score for MVCT- and kVCT-based radiomics were 0.667 and 0.685, respectively. The C-indices of 6 clinical factors were 0.538–0.622. The 3-year OS was significantly different between high- and low-risk groups according to the MVCT-based rad score (50% vs. 83%; p < 0.01).

**Conclusions:**

Our results suggested that MVCT-based radiomics had stronger prognostic power than any single clinical factor and was a useful prognostic factor when predicting OS in HNSCC patients.

**Supplementary Information:**

The online version contains supplementary material available at 10.1186/s12880-023-01055-w.

## Background

Radiomics is an emerging field that converts imaging data into a high mineable feature because it is expected that imaging data contains information that reflect underlying pathophysiology [1, 2]. Radiomics is attracting considerable interest due to its predictive power for overall survival (OS), treatment response and probability of occurrence of side effects, e.g., radiation-induced lung injury and acute xerostomia [3–6]. These studies are often examined using kilo-voltage computed tomography (kVCT) images obtained for diagnosis and treatment planning of radiotherapy.

Cone beam computed tomography (CBCT) images, often obtained to verify patient positioning before delivering each fraction of radiotherapy can monitor changes in radiomic features during the treatment course (delta radiomics). Delta radiomics analysis of images of head and neck cancer patients taken at multiple instances during the treatment period may enable prognostic prediction that reflects the treatment’s effect during that time. If this feasibility is confirmed, it may enable personalized patient treatment such as increasing the dose for HNSCC patients with radioresistant tumors. CBCT-based delta radiomic features (“energy” and “inverse difference normalized”) had significant association with OS in patients with lung cancer [7]. Since it is not easy to frequently utilize dedicated CT imaging equipment for obtaining CT images, radiomics with images for patient position verification (e.g., Megavoltage computed tomography (MVCT) or CBCT images) has a great advantage in carrying delta radiomics. MVCT is also performed to verify patient positioning before radiotherapy using TomoTherapy. Additionally, for head and neck squamous cell carcinoma (HNSCC) patients, MVCT images are less affected than CBCT images to metal artifacts caused by denture. Thus, there is great potential for the application of MVCT-based radiomics to delta radiomics for HNSCC patients. However, whether the radiomic features of MVCT images have prognostic power remains unclear as these images contain more noise than CBCT and planning CT images obtained using kilovoltage X-ray. Gu et al. demonstrated a motion effect of the imaging object and MVCT scan parameters on the reproducibility of the MVCT-based radiomic features [8]. To our knowledge, no studies have examined the association between MVCT-based radiomic features and prognostic power for patients with cancer.

Therefore, this study aimed to investigate whether MVCT-based radiomic features have prognostic power for predicting OS in patients with head and neck squamous cell carcinoma by comparing them with kVCT-based radiomics and established clinical factors.

## Methods

### Patients and imaging datasets

In this retrospective study, informed consent was obtained in the form of opt-out on our website with institutional review board approval, we reviewed the data of 159 patients with stage I–IV localized HNSCC treated with radiation alone or chemoradiation with a curative intent using TomoTherapy Hi-Art system (Accuray, WI, USA) at a single Japanese institution between 2012 and 2021. The treatment strategy followed at our institution was as follows: the prescribed dose was 70 Gy in 35 fractions delivered via IMRT technique using 6-MV X-rays. Patients with stage I and II HNSCCs were typically treated using radiation therapy alone, whereas those with locally advanced stage III–IV HNSCCs were treated using concurrent chemoradiotherapy (CCRT). For patients receiving CCRT, triweekly cisplatin was administered during radiotherapy. In all patients, treatment planning CT images and MVCT images were obtained prior to radiotherapy fraction delivery. Patients meeting the following criteria were excluded: (1) those who had received palliative treatment or postoperative radiotherapy and (2) those with a follow-up period < 3 months. A total of 100 patients were included and analyzed in this study. Planning CT images were acquired using the Aquilion LB CT scanner (Canon Medical Systems, Otawara, Japan). For planning CT images, the mAs value and peak tube voltage were 350 mAs and 120 kV, respectively. Images were reconstructed with an axial plane of 512 × 512 matrix and slice thickness of 2 mm (n = 98) or 3 mm (n = 2). Hereafter, planning CT is referred to as “kVCT.” MVCT images were acquired with X-rays at 3.5-MV energy. The stability of MVCT images is ensured through periodic QA based on the report of AAPM Task Goup-148 [9]. The acquisition pitch was “normal” (8 mm/rotation; n = 14) or “coarse” (12 mm/rotation; n = 86) and the reconstruction interval was 3 mm (n = 82), 4 mm (n = 14), or 6 mm (n = 4). Patient data were divided into training and test datasets in the ratio of 70% and 30%, respectively. OS was defined as the time from the first day of treatment until death from any cause. Patients alive at the last known follow-up were censored. Patient characteristics are shown in Table 1. The Brinkman index (BI) was calculated as the number of cigarettes smoked per day multiplied by the number of years of smoking [10]. To examine whether any difference exists between the two groups when training and test sets were divided, the wilcoxon signed rank test was used for continuous variables (age, follow-up period, BI, and tumor volume), and Pearson’s chi-squared test was used for categorical variables (gender, histology, clinical stage, and treatment method).

**Table 1:** Summary of patient characteristics

Characteristic		Overall n = 100	Training n = 70	Test n = 30	P value
Gender	Female	18 (18%)	13 (16%)	5 (17%)	0.82
	Male	82 (82%)	57 (84%)	25 (83%)	
Age (years: mean ± SD)		67.1 ± 9.40	67.7 ± 9.5	65.5 ± 9.2	0.40
Histology	Larynx	11 (11%)	7 (10%)	4 (13%)	0.75
	Oral cavity	1 (1%)	1 (1%)	0 (0%)	
	Mesopharynx	32 (32%)	21(30%)	11 (37%)	
	Hypopharynx	56 (56%)	41 (59%)	15 (50%)	
Clinical stage	I	7 (7%)	4 (6%)	3 (10%)	0.76
	II	17 (17%)	11 (16%)	6 (20%)	
	III	21 (21%)	16 (23%)	5 (17%)	
	IV	55 (55%)	39 (56%)	16 (53%)	
Treatment method	Radiation only	30 (30%)	20 (29%)	10 (33%)	0.63
	Chemo-radiation	70 (70%)	50 (71%)	20 (67%)	
Brinkman index (mean ± SD)		747.9 ± 503.7 (unknown: n = 7)	733.7 ± 560.1 (unknown: n = 4)	781.1 ± 343.7 (unknown: n = 3)	0.49
Tumor volume (cm^3^: mean ± SD)		19.5 ± 31.5	19.5 ± 35.2	19.4 ± 21.0	0.25
Follow-up (Month: median)		33	37	32	0.77

### Gross tumor volume propagation

**Fig. 1:**
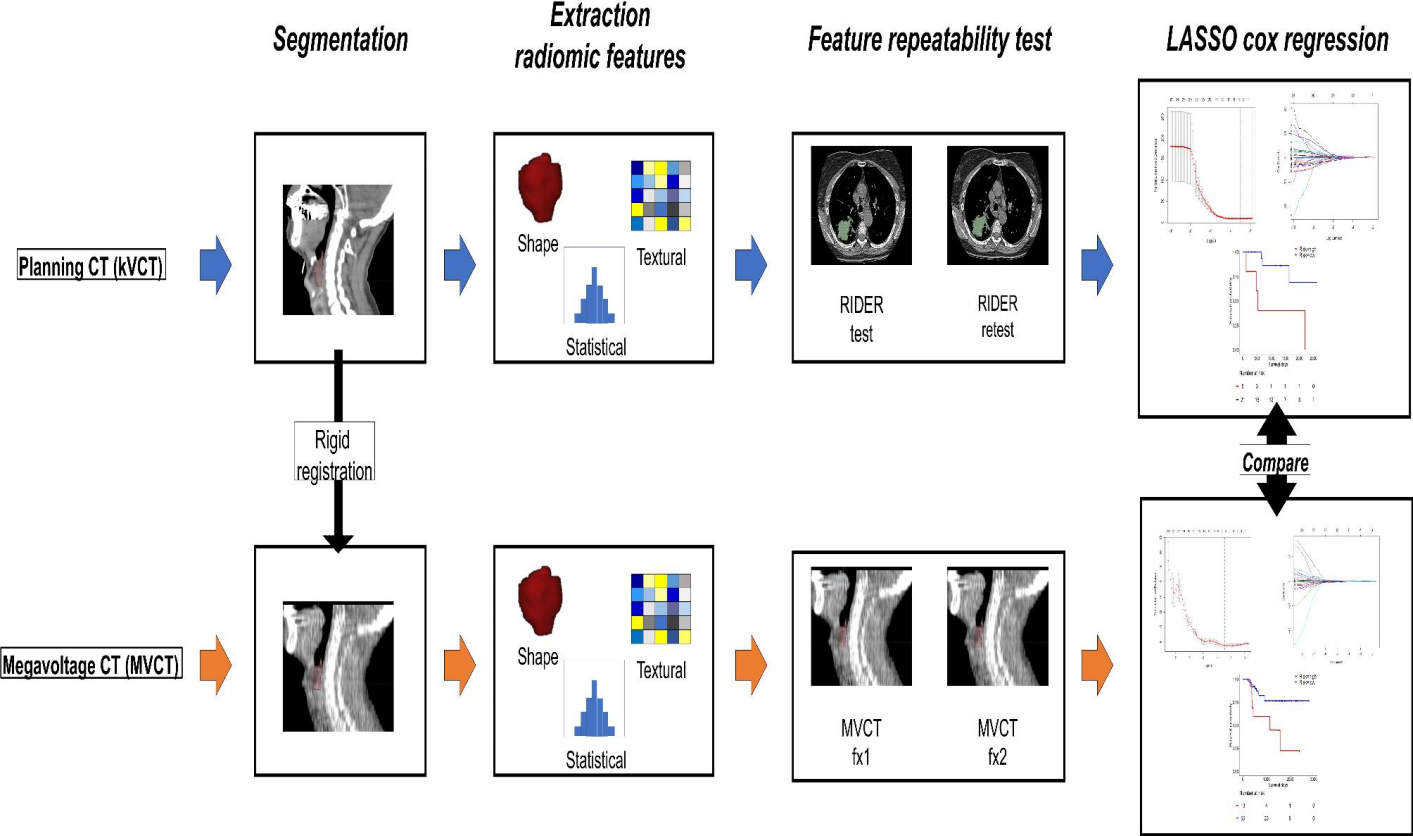
Workflow for comparing MVCT- and kVCT-based radiomics

Figure 1 shows the workflow of this study. The first step was to propagate the gross tumor volume (GTV) of the primary tumor from kVCT to MVCT images using rigid registration. The clinical target volume (CTV) contour of patients with HNSCC often extends to the body surface and is affected to a large extent by the registration error. Thus, in this study, radiomic features were extracted from GTV, and not CTV. GTV was manually delineated on kVCT images for clinical treatment planning by an experienced radiation oncologist. Post-processing was performed on GTV contours. First, the contour slice affected by the metal artifact was removed. In addition, to prevent the inclusion of air and bone in the contour, GTV contours were post-processed to include only soft tissues in the range of − 20 to 180 HU [11]. The GTV contours were then propagated from kVCT to MVCT images of the first fraction using auto rigid registration in MIM Maestro (MIM software, OH, USA). If a registration error was identified at this point by the medical physicist, the registration was manually corrected.

### Radiomic feature extraction

The second step was to extract radiomic features from MVCT and kVCT images. Radiomic features were calculated using an open source radiomic library (Pyradiomics; Harvard Medical School, MA, USA). The imaging biomarker standardization initiative (IBSI), which provides the validated definition and feature benchmark, was established for standardizing radiomic analysis. Pyradiomics is supported by IBSI [12, 13]. We examined 107 radiomic features from MVCT and kVCT images, respectively. These features were divided into 24 Gy-level co-occurrence matrices (GLCMs), 18 first-order statistics features (First order), 16 Gy-level run length matrices (GLRLMs), 14 Gy-level size zone matrices (GLSZMs), 4 neighborhood gray-tone difference matrices (NGTDMs), and 14 Gy-level dependence matrices (GLDMs). In this study, the log filter- and wavelet filter-based radiomic features were excluded from the analysis to compare the prognostic power of kVCT- and MVCT-based radiomics when using simple features. When calculating radiomic features, the number of bins was set as 25. For MVCT and kVCT datasets, a discrepancy in reconstruction setting was observed. Therefore, all images were resampled into equal voxel sizes of 1 × 1 × 1 mm^3^.

### Selection of repeatable radiomic features

The third step was to select repeatable radiomic features. For MVCT-based radiomics, repeatable features were selected using concordance correlation coefficients (CCC) computed between pairs of feature values extracted from MVCT images of the first and second treatment fractions in 10 patients. Due to the absence of public dataset for test-retest analysis of MVCT images, test-retest datasets should be prepared individually. Therefore, we assumed that treatment changes were negligible between the first and second treatment fractions and used MVCT images of the first and second treatment fractions to select repeatable MVCT-based radiomic features. This method has also been used in previous studies using CBCT-based radiomics [7]. These 10 patients were among the 159 patients reviewed in this study but were independent from the 100 patients used for the prognostic analysis. These 10 patients underwent MVCT imaging for the first two fractions using the same scan parameters (the acquisition pitch and reconstruction interval were “Coarse” and 3 mm, respectively). The MVCT imaging scan parameters of 83 out of 100 patients were the same as above. Thus, the scan parameters of most patients (83/100 patients) used in the prognostic prediction analysis and those of the MVCT images used in the reproducibility test were the same. MVCT images of 10 patients were resampled to 1 × 1 × 1 mm^3^. For kVCT-based radiomics, repeatable features were selected using CCC computed with test-retest RIDER datasets on the Cancer Imaging Archive (TCIA, http://www.cancerimagingarchive.net/) []. Test-retest RIDER dataset included 32 patients. Two thoracic CT scans were performed for each patient using the same scanner within a 15-min interval with the same CT acquisition protocol [14]. CCCs were calculated using data of a total of 31 patients because only 1 patient did not have data from two scans. CT images of all patients were resampled to 1 × 1 × 1 mm^3^ because having different voxel sizes can induce bias in CCC results [15]. The disease site was different between this study’s dataset and the RIDER dataset. Reportedly, differences in the disease site and imaging protocol may affect the reproducibility of radiomic feature evaluations [16]. However, Traverso et al. reported that high repeatability and reproducibility features should be used to reduce the risk of false positive associations in the created model [17]. Thus, the RIDER dataset was used for the kVCT-based radiomics analysis of patients with HNSCC to reduce the non-repeatable features derived from kVCT images as much as possible. The repeatability evaluation methods of kVCT- and MVCT-based radiomics are different in terms of acquisition timing and disease site. Thus, to increase the reliability of this study, in addition to the prognostic model created with repeatability test, the prognostic model created without feature reduction by repeatability tests on both the kVCT- and MVCT-based radiomic features was analyzed. A threshold CCC of > 0.85 was used to select repeatable features in MVCT- and kVCT-based radiomics [18, 19].

### Rad score construction

The fourth step was to calculate rad score for each patient using LASSO Cox regression. Based on selected repeatable features, a LASSO Cox regression analysis was performed on the training dataset to select the most useful prognostic combination of radiomic features for predicting OS. This analysis has often been used for radiomics analysis [18–20]. Normalization was performed for the radiomic feature values before LASSO Cox regression analysis. The LASSO regression used for feature selection is performed using the following formula:

$$\widehat {\beta {\rm{ }}}\,{\rm{ = }}\,{\rm{argmin}}\left\{ {\parallel {\rm{y - X}}\parallel _{\rm{2}}^{\rm{2}}{\rm{ + }}\lambda \parallel \beta {\parallel _{\rm{1}}}} \right\},$$

where y is the objective variable, x is the individual feature value, β is the LASSO coefficient, and λ is the regulation weight. The optimal λ that minimized the partial likelihood deviance was explored using the 10-fold cross-validation. The coefficients were then estimated by regression when using the optimal λ. Coefficients of irrelevant radiomic features were set to exactly zero. When using the optimal λ, features with nonzero coefficients were used for fitting the Cox regression model. These features were further integrated into a radiomic signature. An individualized rad score was calculated from a linear combination of multiple selected features, weighted by their respective coefficients (β):

$${\rm{Rad}}\,{\rm{score = }}\,\sum\limits_{{\rm{i = 1}}}^{\rm{n}} {{\beta _{\rm{i}}}} \cdot {\rm{featur}}{{\rm{e}}_{\rm{i}}}$$

### Statistical analysis

The relationship between the rad score and OS was assessed using Kaplan–Meier survival analysis. Patients in both sets were stratified into the high- and low-risk groups based on the rad score. Then the optimal threshold of the rad score was calculated from the training set using “suv_cutpoint” function of the “survminer” package [21]. Because the Kaplan − Meier curve in this study showed that the difference between the survival curves of the high- and low-risk groups over time, the log-rank test was used to quantify significant differences between the high- and low-risk groups. Hazard ratio and C-index were also used to assess the performance of LASSO Cox regression with rad score. C-index was validated on 100 bootstrapped sets constructed through random resampling of the test set [22]. Then, the 95% confidence intervals (CI) were estimated. The univariate predictive performance of clinical predictors was also assessed in the same manner. Clinical predictors included age, stage, treatment method, histology, BI, and tumor volume. Spearman correlation coefficients were calculated to analyze the interchangeability between the MVCT- and kVCT-based radiomic features. A threshold in spearman correlation coefficients of > 0.85 was used to confirm interchangeable features between the MVCT- and kVCT-based radiomics [23]. In this study, the Shapiro–Wilk normality test showed that the feature data set did not demonstrate a normality distribution; therefore, the Spearman correlation test i.e., the nonparametric test, was used.

Statistical analysis was performed using the R software version 3.6.3 (http://www.R-project.org). The R package “survival” and “glmnet” were used for the LASSO Cox regression modeling and survival analysis. A *P*-value of < 0.05 was considered significant.

## Results

A total of 37 and 26 radiomic features had CCC of ≥ 0.85 in MVCT- and kVCT-based radiomic features, respectively. All repeatable radiomic features are listed in Tables S1 and S2. Three of the 37 MVCT-based radiomic features and two of the 26 kVCT-based radiomic features were selected using LASSO. Then the rad score of MVCT- and kVCT-based selected radiomic features was calculated for each patient using the following formula:

$$\begin{array}{l}{\rm{MVCT - based}}\,{\rm{rad}}\,{\rm{score}}\\{\rm{ = }}\,{\rm{GLSZM - GrayLevelNonUniformity}}\, \times {\rm{(1}}{\rm{.76 }} \times {\rm{1}}{{\rm{0}}^{{\rm{ - 1}}}}{\rm{)}}\,{\rm{ + }}\\{\rm{GLSZM - ZoneVariance}}\, \times {\rm{(2}}{\rm{.04}} \times {\rm{1}}{{\rm{0}}^{{\rm{ - 2}}}}{\rm{) + }}\\{\rm{Shape - }}\,{\rm{Maximum2DDiameterRow}}\, \times \left( {{\rm{4}}{\rm{.38}} \times {\rm{1}}{{\rm{0}}^{{\rm{ - 2}}}}} \right),\end{array}$$

$$\begin{array}{l}{\rm{kVCT - based}}\,{\rm{rad}}\,{\rm{score}}\\{\rm{ = }}\,{\rm{GLDM - DependenceNonUniformity}}\, \times {\rm{(1}}{\rm{.23}} \times {\rm{1}}{{\rm{0}}^{{\rm{ - 1}}}}{\rm{)}}\,{\rm{ + }}\\{\rm{Shape - Maximum2DDiameterRow}}\, \times {\rm{(1}}{\rm{.72}} \times {\rm{1}}{{\rm{0}}^{{\rm{ - 1}}}}{\rm{)}}.\end{array}$$

**Fig. 2:**
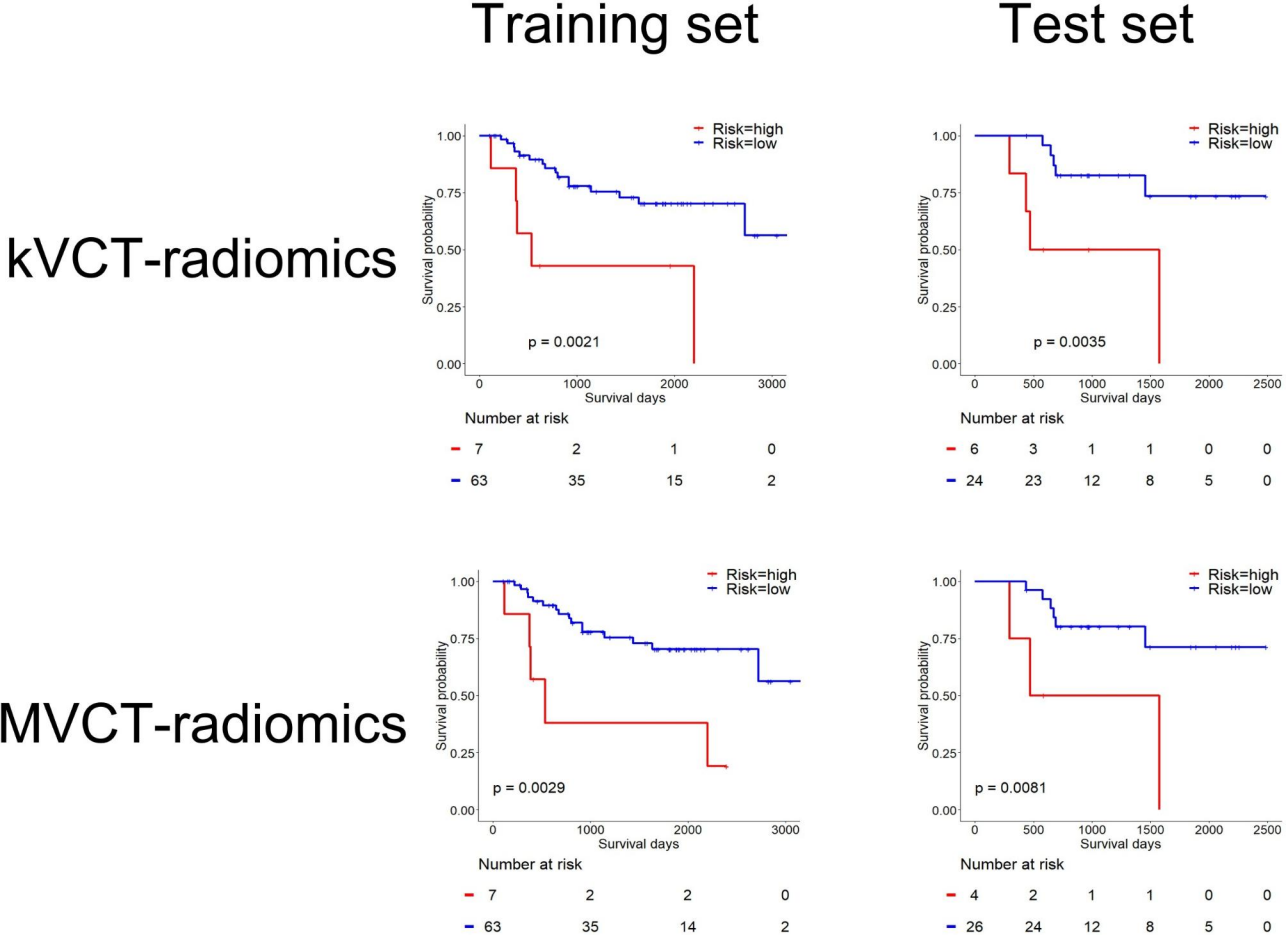
Kaplan–Meier survival curves for low- and high-risk groups based on the rad score. The tick marks represent censored observations

**Fig. 3:**
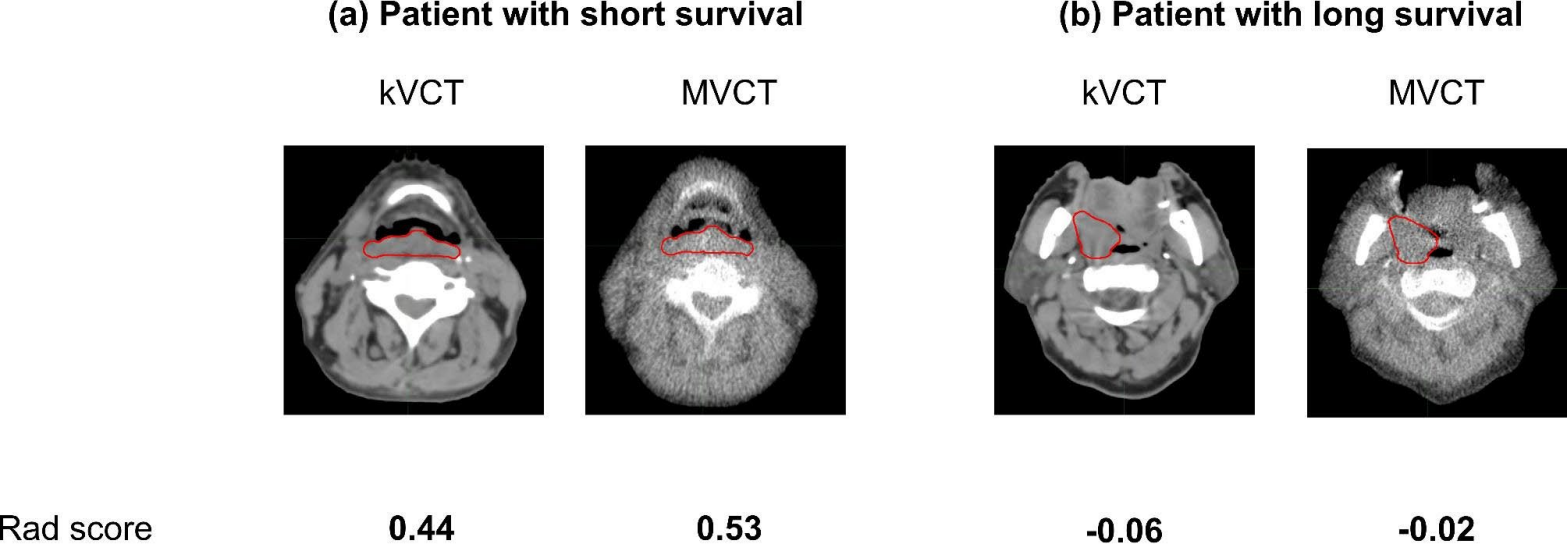
Two representative cases of kVCT and MVCT: a patient with short survival (**a**) and a patient with long survival (**b**). The values of the rad score are shown under each image

Figure 2 shows that patients in both sets were divided into high- and low-risk groups based on the rad score. The optimal cutoff values for MVCT- and kVCT-based rad scores were 1.57 × 10^− 1^ and 1.85 × 10^− 1^, respectively. Univariate and rad score-based analyses of OS in training and test datasets are shown in Table 2. C-indices of the rad score with MVCT- and kVCT-based radiomics were 0.667 and 0.685, respectively. In addition, C-indices of the rad score with MVCT- and kVCT-based radiomics were higher than the established clinical predictors. In rad score-based analysis without feature reduction by the repeatability tests, the kVCT-based radiomics could not divide patients into two groups with Kaplan-Meier analysis. (Table S3 and Figure S1). Figure 3 shows two representative cases with kVCT and MVCT images of patients classified by rad score into high-risk group and low risk group. The survival times of the two patients were different (297 days vs. >961 days). The rad score can be used to classify each patient based on survival length (short and long). Table 3 shows the interchangeable features between the kVCT and MVCT-based radiomics. Table S4 shows the all the spearman correlation coefficients between the kVCT and MVCT-based radiomic features. A total of 23 out of 107 (21.4%) radiomic features showed interchangeability between the kVCT and MVCT-based radiomics. Spearman correlation coefficients between MVCT- and kVCT-based three radiomic features selected by LASSO at MVCT-based radiomic analysis were 0.87 (GLSZM-GrayLevelNonUniformity), 0.92 (GLSZM-ZoneVariance), and 1.00 (Shape- Maximum2DDiameterRow).

**Table 2:** Univariate and rad score-based analyses of overall survival in the training and test sets

Feature		Training set		Test set	
		C-index	p value (log-rank test)	C-index (95%CI)	p value (log-rank test)
kVCT-based radiomics	Rad score	0.558	< 0.01	0.685 (0.679–0.691)	< 0.01
MVCT-based radiomics	Rad score	0.562	< 0.01	0.667 (0.661–0.673)	< 0.01
Clinical factor	Age	0.573	< 0.01	0.583 (0.579–0.588)	0.94
	Stage	0.576	0.70	0.538 (0.535–0.541)	0.40
	Treatment method	0.600	0.32	0.622 (0.617–0.627)	0.08
	Histology	0.588	0.20	0.576 (0.572–0.581)	0.20
	Brinkman index	0.504	0.08	0.571 (0.566–0.576)	0.18
	Tumor volume	0.551	< 0.01	0.621 (0.615–0.627)	< 0.01

CI: Confidence interval

**Table 3:** Interchangeable features between the kVCT and MVCT-based radiomics

Feature		Spearman correlation coefficients
Shape	VoxelVolume	0.998
Shape	Maximum3DDiameter	0.998
Shape	MeshVolume	0.998
Shape	MajorAxisLength	0.998
Shape	Maximum2DDiameterSlice	0.998
Shape	SurfaceArea	0.998
Shape	LeastAxisLength	0.997
Shape	MinorAxisLength	0.997
Shape	Maximum2DDiameterColumn	0.997
Shape	Maximum2DDiameterRow	0.997
Shape	Flatness	0.992
GLDM	DependenceNonUniformity	0.988
Shape	Elongation	0.984
NGTDM	Coarseness	0.977
Shape	SurfaceVolumeRatio	0.974
Shape	Sphericity	0.972
GLDM	GrayLevelNonUniformity	0.970
GLRLM	GrayLevelNonUniformity	0.967
GLRLM	RunLengthNonUniformity	0.930
GLSZM	ZoneVariance	0.923
GLSZM	LargeAreaEmphasis	0.921
GLSZM	GrayLevelNonUniformity	0.870
GLSZM	LargeAreaHighGrayLevelEmphasis	0.853

Abbreviation: GLDM = gray level dependence matrix,

GLCM = gray level co-occurance matrix, GLRLM = gray level run length matrix,

GLSZM = gray level size zone matrix,

NGTDM = neighborhood gray tone difference matrix,

## Discussion

To the best of our knowledge, this is the first study to evaluate the feasibility of using MVCT-based radiomic features to predict the prognosis of patients with HNSCC. Our results showed the potential of MVCT-based radiomics for predicting the OS of patients with HNSCC and the predictive performance of MVCT-based radiomics was comparable with kVCT-based radiomics (C-index: 0.667 vs. 0.685). The prognostic model with MVCT-based radiomics had a higher C-index than prognostic model with established clinical predictors, including tumor volume, age, clinical stage, treatment method (RT alone/CCRT), histology, and BI.

kVCT-based radiomics is a useful prognostic modality for HNSCC patients based on published studies (C-index: 0.61–0.69) [3, 24]. Our results showed that MVCT-based radiomics had the comparable prediction accuracy as kVCT-based radiomics (C-index: 0.667). This may be because they captured similar image features of tumor. MVCT-based radiomic features selected by LASSO and used in the analysis were GLSZM-GrayLevelNonUniformity, GLSZM-ZoneVariance, and Shape-Maximum2DDiameterRow. The Spearman correlation coefficients between the MVCT- and kVCT-based these three radiomic features used for OS prediction at MVCT-based radiomic analysis were > 0.87, confirming interchangeability.

We have outlined several advantages of MVCT images. First, MVCT scans are considered to have less streak and metal artifact as well as less limiting scanning range and field of view than CBCT scans, indicating that the former are advantageous in radiomics analysis compared to the latter [25]. In particular the MVCT image which is less likely to occur metal artifacts is useful for the radiomics analysis of the HNSCC patient because the HNSCC patient often wear dentures (in this study, the percentage of denture-wearing patients was 20%). Second, MVCT-based radiomics has the potential to create a versatile prognostic model. For TomoTherapy, only few scan parameters (acquisition pitch and reconstruct interval) exist when acquiring MVCT images, and a previous study demonstrated negligible differences in image noise (range, 2.32–2.42%) and image uniformity (range, 99.54–99.83%) among the different acquisition pitches and reconstruct intervals [26]. Therefore, by unifying scan parameters, extracting MVCT-based radiomic features that are reproducible across multiple institutions may be possible. Third, the MVCT-based radiomics can be applied to delta radiomics analysis as it allows us to provide individualized radiotherapy that can adapt to the treatment response of each patient. For instance, it may be possible to replan the treatments that improve the toxicity of normal tissues and the tumor control rate, following the results of delta radiomics. However, since MVCT images obtained for radiotherapy positioning are used for delta radiomics analysis, additional patient exposure or biopsies predicting prognosis is unnecessary, which is another advantage of this procedure. This study has some limitations. First, the study dataset was heterogeneity cohort including patients with stage I–IV localized HNSCC, and the treatment regimen is also mixed. Second, to select the repeatable features, repeatable kVCT-based radiomic features were selected using the dataset with different disease sites from the MVCT datasets and kVCT analysis data set. Ideally, it is better to use the same pipeline to select the repeatable radiomic features. However, compared to the prediction results without feature reduction, the model created after feature reduction had a higher C-index, and could detect the difference in survival rates, suggesting that feature reduction of this study worked well to avoid over-fitting of the prediction model. Third, the GTV contours were propagated to MVCT fx1 from the planning CT (kVCT) images using rigid registration by bone matching. This method assumes that there are few major anatomical changes due to the short period between the planning CT (kVCT) and MVCT of the first radiotherapy.

## Conclusions

We evaluated the prognostic power of MVCT-based radiomic features. Our results showed the prediction accuracy of the MVCT-based radiomics was higher than the established any single clinical factor and comparable to the kVCT-based radiomics. Therefore, MVCT-based radiomics can be used for predicting OS in patients with HNSCC. The results of this study imply that applying MVCT images to delta radiomics analysis may allow a better prognosis prediction of the treatment than conventional methods. If its feasibility is verified, it may become a tool for patient-personalized medicine. We will examine delta radiomics utilizing MVCT images following the present results.

## Electronic supplementary material

Below is the link to the electronic supplementary material.

**Additional file 1**: **Fig S1**. Kaplan–Meier survival curves for low- and high-risk groups based on the rad score calculated without feature reduction by repeatability test. The tick marks represent censored observations. **Table S1**. List of repeatable MVCT-based radiomic features and their CCC value. **Table S2**. List of repeatable kVCT-based radiomic features and their CCC value. **Table S3**. Rad score-based analyses of the overall survival in training and test sets without feature reduction by repeatability test. **Table. S4**. Spearman correlation coefficients between the kVCT and MVCT-based radiomic features. Bold type indicates features with interchangeability.

## Data Availability

The datasets generated and/or analyzed during the current study are not publicly available because the use of patient data (medical images and reports) other than by us is not approved by the patients, but are available from the corresponding author on reasonable request.

## Abbreviations

CBCT: Cone-beam computed tomography
CCC: Concordance correlation coefficients
CCRT: Concurrent chemoradiotherapy
CTV: Clinical target volume
First order: First-order statistics features
GLCM: Gray-level co-occurrence matrices
GLDM: Gray-level dependence matrices
GLRLM: Gray-level run length matrices
GLSZM: Gray-level size zone matrices
GTV: Gross tumor volume
HNSCC: Head and neck squamous cell carcinoma
IBSI: The imaging biomarker standardization initiative
kVCT: Kilovoltage CT
MVCT: Megavoltage computed tomography
NGTDM: Neighborhood gray-tone difference matrices
NSCLC: Non–small-cell lung cancer
OS: Overall survival
pCT: Planning CT image
TCIA: The Cancer Imaging Archive
