# Supplementary material for: Evaluation of the MVCT-based radiomic features as prognostic factor in patients with head and neck squamous cell carcinoma
Source: BMC Med Imaging. 2023 Aug 1;23:102. doi: 10.1186/s12880-023-01055-w (PMC10391970; doi:10.1186/s12880-023-01055-w)
Supplement: Supplementary file 1 — Additional file 1: Fig S1. Kaplan–Meier survival curves for low- and high-risk groups based on the rad score calculated without feature reduction by repeatability test. The tick marks represent censored observations. Table S1. List of repeatable MVCT-based radiomic features and their CCC value. Table S2. List of repeatable kVCT-based radiomic features and their CCC value. Table S3. Rad score-based analyses of the overall survival in training and test sets without feature reduction by repeatability test. Table. S4. Spearman correlation coefficients between the kVCT and MVCT-based radiomic features. Bold type indicates features with interchangeability. [file 12880_2023_1055_MOESM1_ESM.pdf]

Supplement materials

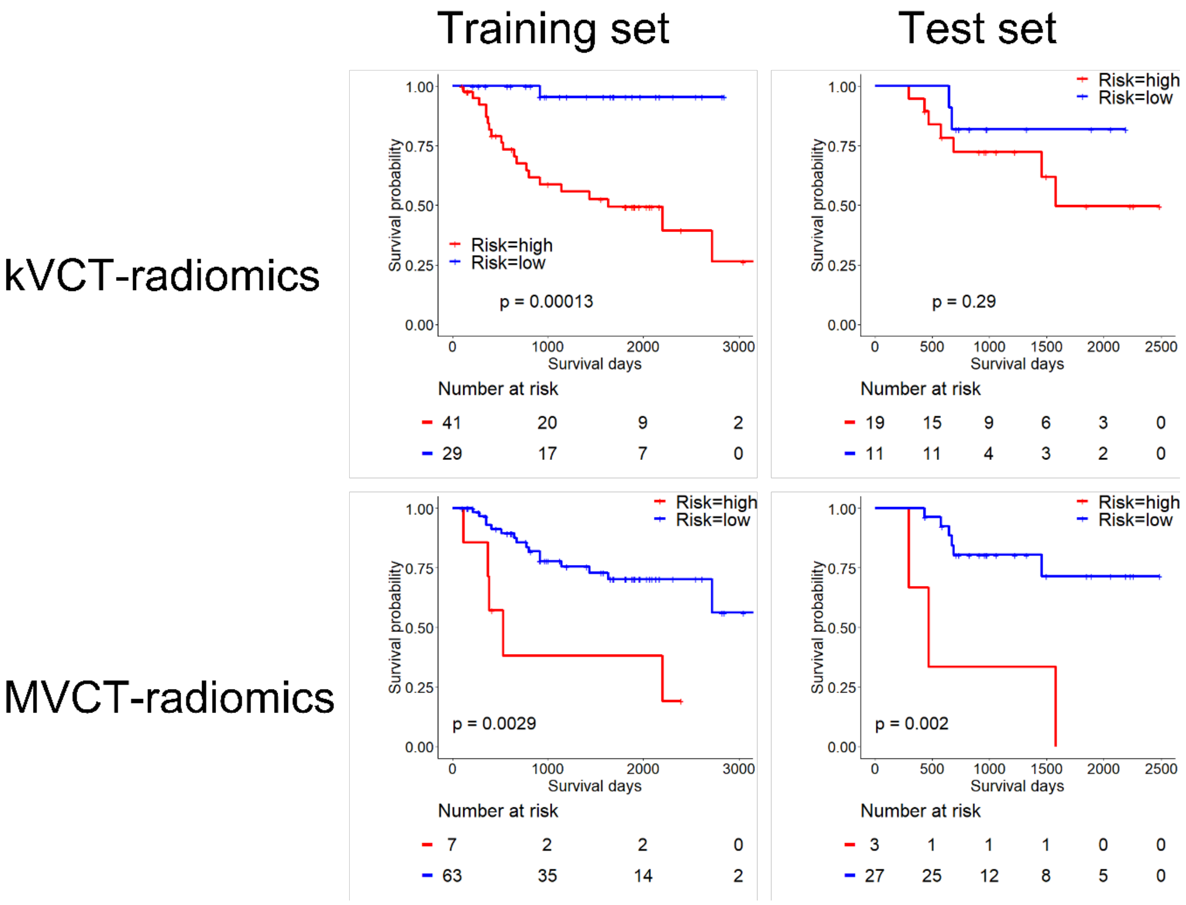

Fig S1. Kaplan–Meier survival curves for low- and high-risk groups for OS based on the rad score calculated without feature reduction by repeatability test. The tick marks represent censored observations.

Table. S1. List of repeatable MVCT-based radiomic features and their CCC value.

	Feature	CCC value
shape	MeshVolume	0.9996
shape	VoxelVolume	0.9996
GLDM	GrayLevelNonUniformity	0.9995
GLRLM	GrayLevelNonUniformity	0.9994
GLRLM	RunLengthNonUniformity	0.9992
GLDM	DependenceNonUniformity	0.9991
shape	Maximum2DDiameterSlice	0.9988
GLSZM	GrayLevelNonUniformity	0.9969
GLSZM	ZoneVariance	0.9966
GLSZM	LargeAreaEmphasis	0.9966
shape	MinorAxisLength	0.9961
shape	SurfaceArea	0.9933
shape	LeastAxisLength	0.9912
shape	Maximum3DDiameter	0.9759
shape	SurfaceVolumeRatio	0.9739
shape	Maximum2DDiameterRow	0.9734
shape	Maximum2DDiameterColumn	0.9733
shape	Flatness	0.9587
GLRLM	RunVariance	0.9562
GLSZM	SizeZoneNonUniformity	0.9556
Firstorder	Energy	0.9456
Firstorder	TotalEnergy	0.9456

NGTDM	Strength	0.9426
GLRLM	LongRunEmphasis	0.9419
shape	MajorAxisLength	0.9331
NGTDM	Coarseness	0.9240
GLDM	SmallDependenceLowGrayLevelEmphasis	0.9137
GLDM	LargeDependenceEmphasis	0.9107
GLRLM	ShortRunLowGrayLevelEmphasis	0.9025
GLSZM	LowGrayLevelZoneEmphasis	0.9008
GLRLM	LowGrayLevelRunEmphasis	0.8984
GLDM	LowGrayLevelEmphasis	0.8906
GLDM	LargeDependenceLowGrayLevelEmphasis	0.8809
GLDM	DependenceVariance	0.8721
GLRLM	LongRunLowGrayLevelEmphasis	0.8712
GLRLM	RunPercentage	0.8676
GLRLM	ShortRunEmphasis	0.8596

Abbreviation: GLDM = gray level dependence matrix, GLRLM = gray level run length matrix, GLSZM = gray level size zone matrix, NGTDM = neighborhood gray tone difference matrix, CCC = concordance correlation coefficient

Table. S2. List of repeatable kVCT-based radiomic features and their CCC value.

Feature		CCC value
shape	MeshVolume	0.9819
shape	VoxelVolume	0.9806
GLSZM	GrayLevelNonUniformity	0.9784
GLRLM	RunLengthNonUniformity	0.9783
GLRLM	GrayLevelNonUniformity	0.9751
GLDM	GrayLevelNonUniformity	0.9701
GLDM	DependenceNonUniformity	0.9689
GLSZM	SizeZoneNonUniformity	0.9528
NGTDM	Busyness	0.9478
shape	MinorAxisLength	0.9344
shape	MajorAxisLength	0.9292
Firstorder	Maximum	0.9227
Firstorder	Range	0.9214
shape	SurfaceArea	0.9123
shape	LeastAxisLength	0.8946
shape	Maximum2DDiameterRow	0.8906
NGTDM	Complexity	0.8906
GLSZM	LargeAreaLowGrayLevelEmphasis	0.8801
Firstorder	Energy	0.8717
Firstorder	TotalEnergy	0.8717
shape	Maximum2DDiameterSlice	0.8682
GLSZM	ZoneEntropy	0.8677
GLSZM	LargeAreaEmphasis	0.8593

GLSZM	ZoneVariance	0.8592
GLDM	LargeDependenceLowGrayLevelEmphasis	0.8557
shape	Elongation	0.853

Abbreviation: GLDM = gray level dependence matrix, GLRLM = gray level run length matrix, GLSZM = gray level size zone matrix, NGTDM = neighborhood gray tone difference matrix, CCC = concordance correlation coefficient

Table. S3. Rad score-based analyses of the overall survival in training and test sets without feature reduction by repeatability test.

Feature		Training set		Test set	
		C-index	p value (log-rank test)	C-index	p value (log-rank test)
kVCT-based radiomics	Rad score	0.748	< 0.01	0.647	0.29
MVCT-based radiomics	Rad score	0.572	< 0.01	0.647	< 0.01

Table. S4. Spearman correlation coefficients between the kVCT and MVCT-based radiomic features. Bold type indicates features with interchangeability.

	Feature	Spearman correlation coefficients
**Shape**	**VoxelVolume**	**0.998**
**Shape**	**Maximum3DDiameter**	**0.998**
**Shape**	**MeshVolume**	**0.998**
**Shape**	**MajorAxisLength**	**0.998**
**Shape**	**Sphericity**	**0.972**
**Shape**	**LeastAxisLength**	**0.997**
**Shape**	**Elongation**	**0.984**
**Shape**	**SurfaceVolumeRatio**	**0.974**
**Shape**	**Maximum2DDiameterSlice**	**0.998**
**Shape**	**Flatness**	**0.992**
**Shape**	**SurfaceArea**	**0.998**
**Shape**	**MinorAxisLength**	**0.997**
**Shape**	**Maximum2DDiameterColumn**	**0.997**
**Shape**	**Maximum2DDiameterRow**	**0.997**
GLDM	GrayLevelVariance	0.297
GLDM	HighGrayLevelEmphasis	0.288
GLDM	DependenceEntropy	0.324
**GLDM**	**DependenceNonUniformity**	**0.988**
**GLDM**	**GrayLevelNonUniformity**	**0.970**
GLDM	SmallDependenceEmphasis	0.684
GLDM	SmallDependenceHighGrayLevelEmphasis	0.270
GLDM	DependenceNonUniformityNormalized	0.132
GLDM	LargeDependenceEmphasis	0.634

GLDM	LargeDependenceLowGrayLevelEmphasis	0.290
GLDM	DependenceVariance	0.433
GLDM	LargeDependenceHighGrayLevelEmphasis	0.389
GLDM	SmallDependenceLowGrayLevelEmphasis	0.698
GLDM	LowGrayLevelEmphasis	0.359
GLCM	JointAverage	0.286
GLCM	SumAverage	0.286
GLCM	JointEntropy	0.413
GLCM	ClusterShade	0.197
GLCM	MaximumProbability	0.343
GLCM	Idmn	0.613
GLCM	JointEnergy	0.410
GLCM	Contrast	0.387
GLCM	DifferenceEntropy	0.438
GLCM	InverseVariance	-0.326
GLCM	DifferenceVariance	0.398
GLCM	Idn	0.571
GLCM	Idm	0.410
GLCM	Correlation	-0.090
GLCM	Autocorrelation	0.287
GLCM	SumEntropy	0.381
GLCM	MCC	0.091
GLCM	SumSquares	0.291
GLCM	ClusterProminence	0.197
GLCM	Imc2	-0.156
GLCM	Imc1	-0.355
GLCM	DifferenceAverage	0.425
GLCM	Id	0.413
GLCM	ClusterTendency	0.242

Firstorder	InterquartileRange	0.359
Firstorder	Skewness	-0.040
Firstorder	Uniformity	0.372
Firstorder	Median	0.016
Firstorder	Energy	0.793
Firstorder	RobustMeanAbsoluteDeviation	0.378
Firstorder	MeanAbsoluteDeviation	0.388
Firstorder	TotalEnergy	0.793
Firstorder	Maximum	0.349
Firstorder	RootMeanSquared	0.016
Firstorder	90Percentile	0.000
Firstorder	Minimum	0.295
Firstorder	Entropy	0.409
Firstorder	Range	0.386
Firstorder	Variance	0.301
Firstorder	10Percentile	0.358
Firstorder	Kurtosis	0.257
Firstorder	Mean	0.118
GLRLM	ShortRunLowGrayLevelEmphasis	0.409
GLRLM	GrayLevelVariance	0.254
GLRLM	LowGrayLevelRunEmphasis	0.379
GLRLM	GrayLevelNonUniformityNormalized	0.369
GLRLM	RunVariance	0.705
**GLRLM**	**GrayLevelNonUniformity**	**0.967**
GLRLM	LongRunEmphasis	0.700
GLRLM	ShortRunHighGrayLevelEmphasis	0.297
**GLRLM**	**RunLengthNonUniformity**	**0.930**
GLRLM	ShortRunEmphasis	0.607
GLRLM	LongRunHighGrayLevelEmphasis	0.379

GLRLM	RunPercentage	0.649
GLRLM	LongRunLowGrayLevelEmphasis	0.259
GLRLM	RunEntropy	-0.114
GLRLM	HighGrayLevelRunEmphasis	0.291
GLRLM	RunLengthNonUniformityNormalized	0.593
GLSZM	GrayLevelVariance	0.230
**GLSZM**	**ZoneVariance**	**0.923**
GLSZM	GrayLevelNonUniformityNormalized	0.144
GLSZM	SizeZoneNonUniformityNormalized	0.413
GLSZM	SizeZoneNonUniformity	0.842
**GLSZM**	**GrayLevelNonUniformity**	**0.870**
**GLSZM**	**LargeAreaEmphasis**	**0.921**
GLSZM	SmallAreaHighGrayLevelEmphasis	0.290
GLSZM	ZonePercentage	0.694
GLSZM	LargeAreaLowGrayLevelEmphasis	0.596
**GLSZM**	**LargeAreaHighGrayLevelEmphasis**	**0.853**
GLSZM	HighGrayLevelZoneEmphasis	0.312
GLSZM	SmallAreaEmphasis	0.408
GLSZM	LowGrayLevelZoneEmphasis	0.532
GLSZM	ZoneEntropy	0.539
GLSZM	SmallAreaLowGrayLevelEmphasis	0.575
**NGTDM**	**Coarseness**	**0.977**
NGTDM	Complexity	0.353
NGTDM	Strength	0.205
NGTDM	Contrast	0.617
NGTDM	Busyness	0.433

Abbreviation: GLDM = gray level dependence matrix, GLCM = gray level co-occurance matrix, GLRLM = gray level run length matrix, GLSZM = gray level size zone matrix, NGTDM = neighborhood gray tone difference matrix,
